# Application progress of artificial intelligence in tumor diagnosis and treatment

**DOI:** 10.3389/frai.2024.1487207

**Published:** 2025-01-07

**Authors:** Fan Sun, Li Zhang, Zhongsheng Tong

**Affiliations:** ^1^National Clinical Research Center for Cancer, Tianjin Medical University Cancer Institute and Hospital, Tianjin, China; ^2^Tianjin’s Clinical Research Center for Cancer, Tianjin, China; ^3^Key Laboratory of Breast Cancer Prevention and Therapy, Tianjin Medical University, Ministry of Education, Tianjin, China; ^4^Key Laboratory of Cancer Prevention and Therapy, Tianjin, China

**Keywords:** artificial intelligence, tumor, pathological diagnosis, image recognition, precision medicine, therapy

## Abstract

The rapid advancement of artificial intelligence (AI) has introduced transformative opportunities in oncology, enhancing the precision and efficiency of tumor diagnosis and treatment. This review examines recent advancements in AI applications across tumor imaging diagnostics, pathological analysis, and treatment optimization, with a particular focus on breast cancer, lung cancer, and liver cancer. By synthesizing findings from peer-reviewed studies published over the past decade, this paper analyzes the role of AI in enhancing diagnostic accuracy, streamlining therapeutic decision-making, and personalizing treatment strategies. Additionally, this paper addresses challenges related to AI integration into clinical workflows and regulatory compliance. As AI continues to evolve, its applications in oncology promise further improvements in patient outcomes, though additional research is needed to address its limitations and ensure ethical and effective deployment.

## Introduction

**Figure 1:**
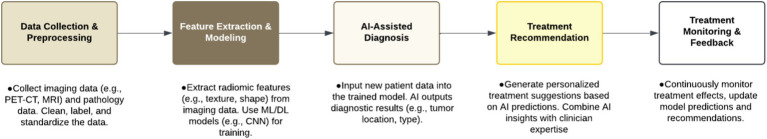
Example of the role of AI in diagnosis and treatment workflows.

The term “artificial intelligence (AI) “was coined at the Dartmouth Summer Symposium in 1956 to describe the former ideas about “thinking machine.” AI refers to a machine’s capacity for autonomous learning, identifying patterns and associations from extensive sample data to make efficient decisions in unfamiliar scenarios (Bhinder et al., 2021). Specifically, machine learning (ML) is a subfield of AI, while deep learning (DL) is a subset of MI. In many fields, such as speech recognition, image classification and text understanding, DL breaks through traditional algorithm and gradually evolves into a novel paradigm that starts from training with representative materials to outputting deliverables via end-to-end modeling. In DL, each layer independently adjusts toward the overall goal, and all layers work synergistically to enhance task accuracy (Yu et al., 2017). With its advancement, AI shows the potential to completely change the medical industry in such domains as follows: assisting doctors in diagnosis to minimize misdiagnosis and missed diagnosis; improving diagnostic efficiency to relax the imbalance between supply and demand of medical resources; providing early warning of disease risk and health consultation services; supporting drug research and development while promoting pharmaceutical productivity; and bettering surgical robots and operational accuracy (Liu and Yu, 2022). Figure 1 illustrates the application pathway of AI in cancer diagnosis and treatment.

The aim of this review is to discuss the latest applications and advancements of artificial intelligence (AI) in tumor diagnosis and treatment, with an emphasis on its transformative impact on traditional diagnostic and therapeutic approaches and future potential. By synthesizing existing research findings, this review examines how AI plays a pivotal role in tumor imaging diagnostics, pathological analysis, and treatment optimization. To ensure a comprehensive and rigorous review, the literature selection was based on the following criteria: (1) only peer-reviewed academic articles and research reports published within the last 10 years were included (2); selected literature must involve applications of AI in tumor diagnosis, treatment, imaging analysis, or pathology. (3) Due to the broad scope and cancer types involved, this review primarily focuses on literature concerning breast, lung, and liver cancers, with emphasis on AI systems that have undergone regulatory approval and entered clinical practice, as well as emerging technologies with significant potential still under investigation. Table 1 presents the currently available AI software used in tumor diagnosis and treatment, along with their current developmental status.

**Table 1:** The available AI software and functions in tumor diagnosis and treatment.

Category	Software name	Description	Developer/organization	Key features	dataset size	Availability status
Pathology diagnosis	PathAI	Utilizes deep learning for automated analysis of pathology images	PathAI (PathAI Inc., Boston, USA)	Enhances diagnostic accuracy for pathologists, reducing misdiagnosis rates	10,000–50,000+ pathology images from diverse sources, including biopsy slides for cancer detection and grading	Available in clinical use
	OncoKB	Analyzes tumor genomic data for drug matching	OncoKB (Memorial Sloan Kettering Cancer Center, New York, USA)	Provides information on clinically relevant genetic alterations to support treatment decisions	Genomic data from over 30,000 tumor samples covering multiple cancer types	Available in clinical use
Imaging diagnosis	Aidoc	AI-driven medical imaging analysis for rapid lesion detection	Aidoc (Aid-oc Medical Ltd., Tel Aviv, Israel)	Instant alert feature to alleviate the workload of radiologists	50,000+ annotated CT and MRI images from clinical cases	Available in clinical use
	Zebra Medical Vision	Automated analysis of medical imaging to support early diagnosis	Zebra Medical Vision (Zebra Medical Ltd., Kibbutz Shefayim, Israel)	Employs deep learning models to improve imaging diagnostic accuracy	Over 1 million medical images, including X-rays, CT scans, and MRI, across multiple health systems	Available in clinical use
	Google DeepMind	Deep learning algorithms for analyzing medical images to assist in diagnosis	Google DeepMind (Google, LLC, London, UK)	Capable of identifying various pathological conditions with self-learning capabilities	Dataset of 10,000+ annotated medical images for disease identification and feature learning	Research stage; trials ongoing
	Philips IntelliSpace	AI-supported medical imaging k-t platform integrating various analysis tools	Philips (Ro-yal Philips, Amsterda, Netherlans)	Provides real-time data analysis and visualization to support diagnosis across multiple imaging modalities	Imaging data from 20,000+ cases across multiple modalities (CT, MRI, ultrasound)	Available in clinical use
Precision medicine	Tempus	Combines clinical data with genomics to offer personalized treatment	Tempus (Tempus Labs, Inc., Chicago, USA)	Offers real-time data analysis to support clinical trials and patient treatment plans	Over 100,000 clinical cases with combined genomic and clinical data, covering various cancers	Available in clinical use
	Prognos Health	Analyzes data to predict treatment responses and disease progression	Prognos Health (Prognos Health Inc., New York, USA)	Utilizes machine learning algorithms to generate prognostic models	30 million+ anonymized healthcare records, including claims, lab results, and medication data	Research stage; trials ongoing
Clinical trials	C4AI	AI platform for clinical trials, analyzing clinical data	C4AI (C4AI Ltd., Cambridge, UK)	Automates trial design and result analysis to enhance trial efficiency	5,000+ clinical trial records with patient demographics, treatments, and outcomes	Research stage; trials ongoing
Remote healthcare	Biofourmis	Combines remote monitoring with AI analysis to assess patient health status	Biofourmis (Biofourmis Inc., Boston, USA)	Monitors physiological data changes to provide personalized health recommendations	Real-time physiological data from over 10,000 patients using wearable devices	Available in clinical use
Cancer screening	GRAIL	Liquid biopsy technology for early cancer detection using AI analysis	GRAIL (GRAIL Inc., Menlo Park, USA)	Analyzes blood samples to assess the likelihood of various cancers	Over 50,000 blood samples from patients, tested across multiple cancer types	Available in clinical use
	Genius Cervical AI	AI-assisted cervical cancer screening tool that analyzes cytology images	Genius Cervical AI (Hologic Inc., Marlborough, USA)	Provides high-accuracy cervical cell analysis for early detection	200,000+ cervical cytology images from clinical screenings	Available in clinical use
Health management	eHealth	AI-based health monitoring and clinical decision support system	eHealth (eHealth Systems Ltd., Toronto, Canada)	Integrates various health data sources to provide comprehensive health management solutions	Aggregated data from over 10 million health records, including lab tests and electronic health records	Available in clinical use
Clinical decision support	IBM Watson Health	Utilizes natural language processing and machine learning for clinical data analysis	IBM Watson Health (IBM Corporation, Armonk, USA)	Integrates data from multiple databases to support personalized treatment decisions	Access to 1.5 million+ patient records across diverse medical conditions	Available in clinical use

### AI for the diagnosis of tumor

#### Pathological diagnosis

Pathological diagnosis, commonly used in clinical practice, reveals etiology based on natural sciences and serves as the “gold standard” for disease identification. Machine-based oncological pathology is the discovery and classification of tumor-related features in pathological photos to determine its nature, stage and prognosis, aiming to improved objectivity and accuracy in diagnosis. In recent years, artificial intelligence, particularly deep learning (DL), has demonstrated significant potential in foci identification, prognosis prediction, microenvironment characterization, metastasis detection, and other pathological analyses for tumor diagnosis. Pathological AI has been applied in multiple types of tumors, such as breast cancer (Zhu et al., 2019), gastric cancer, nasopharyngeal carcinoma (Qi et al., 2021) and colorectal cancer, mainly for determination of benign or malignant nature (George and Sankaran, 2020), disease staging, staining analysis and early screening.

#### Whole slide imaging (WSI)

This digital pathology platform has been used for conferences, virtual seminars, presentations and educational purpose etc. AI enhances the utility of whole slide imaging (WSI), as AI tools assist in training next-generation pathologists by providing on-demand, standardized, and interactive digital slides that can be shared among multiple users anytime and anywhere (Zarella et al., 2019). WSI systems featuring automation, high speed, and high resolution have demonstrated a significant impact on medical quality assurance (QA), particularly when supported by AI, which readily provides digital slides to pathologists via laboratory information systems or intranets for various QA tasks, such as remote consultation, measuring inter-and intra-observer differences, competency testing, and slide archiving (Bera et al., 2019). Saillard et al. (2020) developed two DL algorithms based on WSI to predict the survival rate of patients with hepatocellular carcinoma underwent surgical excision, with both models superior to the traditional method based on comprehensive scores of all survival-related baseline variables, and they further verified prognostic value of the models in The Cancer Genome Atlas (TCGA) dataset.

#### Tumor infiltrating lymphocyte (TIL)

As an important indicator for prediction of the efficacy and outcome of neoadjuvant chemotherapy, TIL is regarded by a growing number of professionals as an important biomarker for many types of cancer (Le et al., 2020; Zhang et al., 2023). However, its application in prognosis is largely limited, since TIL requires much labor for its standardized quantification relying on pathologists and it may additionally involve immunohistochemical staining that seriously lacks of unified measurement method. The advance of digital pathology and artificial intelligence inspires increasing interests to the development of automatic tools for counting and analysis of TILs in pathological sections. The “DeepTIL” created by Xu Hongming team aims to effectively and efficiently evaluate the TILs distribution in whole slide images post-H&E staining and to automatically conduct analysis for assistance in the prognosis of patients with colorectal cancer (Xu et al., 2022). Saltz et al. (2018) employed neural network that incorporated pathologist’s feedback to automatically detect the spatial organization of TILs in tissue section images of cancer genome maps, and they found that this characterization could predict the prognosis of 13 different cancer subtypes. Image analysis based on DL could detect TILs in testicular germ cell tumors rather objectively and makes TIL a prognostic marker of disease recurrence (Linder et al., 2019). Ao et al. (2022) reported an automated high-throughput microfluidic platform for simultaneously tracking the dynamics of T-cell infiltration and the cytotoxicity in 3D tumor cultures with adjustable matrix. This platform could evaluate the efficacy of each treatment with the help of a TIL analyzer based on clinical data-driven DL method. PathExplore IOP accelerates the characterization of immune phenotypes by quantifying tumor-infiltrating lymphocytes (TILs) and their spatial distribution utilizing routine hematoxylin and eosin (H&E) samples. This innovative product enables researchers to evaluate the spatial arrangements of TILs within the tumor core and periphery, thereby providing insights into the immune-inflamed, desert, or excluded characteristics of the sample (Path, 2024).

### Application of AI in image recognition

Medical imaging provides most of the medical data, but image recognition in routine diagnoses mainly depends on manual analysis. This traditional recognition method is limited by subjective judgments of radiologists; hence misdiagnosis may occur. AI support in image recognition and disease diagnosis may play an important role in lesion discovery and significantly improve diagnostic efficiency: independent and smart extraction and mining of image information for better image recognition; automatic and intelligent interpretation of the images based on DL and simulating human neural network for better analysis. Here are some examples: S-Detect™, a new ultrasound system supplied by Samsung (a South Korea company), significantly improved diagnostic performance of inexperienced radiologists for malignant breast tumors using DL algorithm for lesion classification (Lee et al., 2019); a team of researchers from Harvard Medical School and Beth Israel Deaconess Medical Center tested GPT-4’s performance in diagnosis of challenging medical records, and they found that it provided correct differentiation in 64% of the cases and even best diagnosis in 39% of all (Kanjee et al., 2023).

#### PET/CT

[18F] fluorodeoxyglucose ([18F]FDG) positron emission tomography/computed tomography (PET/CT) has become a primary imaging modality in oncology, utilized for disease diagnosis, staging, re-staging, and monitoring therapeutic outcomes (Hu et al., 2021). A research team has developed an automated system for rapid assessment of [18F] FDG PET/CT image quality. The system’s image quality assessment results show high concordance with the ratings of experienced nuclear medicine physicians, with kappa coefficients indicating strong agreement across different anatomical regions. Additionally, the system-generated objective quality metrics (e.g., SUVmax, SUVmean) meet clinical accuracy standards (Qi et al., 2023). AI-enhanced PET radiomics can predict patient response to neoadjuvant chemotherapy (NAC) (Hathi et al., 2020). Moreover, AI tools applied to PET radiomics enhance the detection and characterization of lymph node and distant metastases, improving staging accuracy for breast cancer patients (Li et al., 2021).

#### Radiomics and radiogenomics

MRI radiomics is one of the most widely used techniques, with T2-weighted imaging (T2WI), diffusion-weighted imaging (DWI), and dynamic contrast-enhanced MRI (DCE-MRI) as the commonly used sequences. Radiogenomics establishes models by extracting a large number of quantitative data from segmented images and correlating image features with pathophysiology and gene expression. With further development and progress of AI, radiogenomics is rapidly becoming a powerful new tool in precision medicine, and it may turn into a critical part in clinical treatment planning and prognosis judgment. Fan et al. (2017) pioneered in using 3D volumetric image features to classify molecular subtypes of breast cancer, and then discussed the relationship between DCE-MRI-based radiomic features and molecular typing of breast cancer; By extracting and analyzing the features in morphology, texture, statistics, dynamics and bilateral difference in DCE-MRI images, they succeeded in differentiation of breast cancers among four different molecular subtypes (luminal A, luminal B, HER2 and basal-like) (Fan et al., 2017). Another team reported that a DCE-MRI-based comprehensive analysis of peritumoral and intratumoral radiological features of patients with invasive HER2+ breast cancer, helps in the identification of inherent molecular tumor subtypes and in the insight to the immune response within the environment around lesions, as well as in the prediction of responses to targeted therapy (Braman et al., 2019).

#### Image segmentation

The segmentation of medical images is essential in representation and visualization of relevant structures in the images. Bhanumurthy and Anne (2014) developed an automatic technique that uses AI to accurately detect cerebral MRI signals and segment the images for neoplastic, atrophic and other abnormal tissues. Since the first image recognition system based on convolution neural network (CNN) was proposed in 1995, breast-tumor segmentation using DL has been employed by many medical imaging applications (Conte et al., 2020). Rahimpour et al. (2023) developed a deep CNN using T1-weighted post-contrast and subtraction magnetic resonance imaging (MRI) techniques for automatic three-dimensional (3D) segmentation of breast tumors; their use of visual ensemble selection provided clinically useful segmentation in 77% of the cases, demonstrating the possibility in reducing radiologists’ manual 3D segmentation labor and in greatly promoting the quantitative research of non-invasive biomarkers from breast MRI. George and Sankaran (2020) introduced a decision-making scheme based on patch class probability (NucDeep + SVM + PD) for image-level classification. They evaluated the proposed framework on the publicly accessible BreaKHis dataset through five random trials, achieving a mean image-level recognition rate of 96.66% ± 0.77%, a specificity of 100%, and a sensitivity of 96.21%. Their proposed NucDeep + FF + SVM model was found to be superior to several existing methods at the time and demonstrated comparable state-of-the-art performance even with low training complexity (George and Sankaran, 2020). Qi et al. (2021) reported their work on nasopharyngeal carcinoma: detection by CNN on computed tomography (CT) scans followed by segmentation from MRI images using multimodal MRI fusion network.

## Application of AI in tumor treatment

### Medical treatment

With the background of a large number of data available from high-throughput sequencing, rapidly developing AI technology has provided oncologists with deeper insights into tumors, ushering in a new era of clinical oncology focused on precision treatment and personalized medicine.

#### Precision medical treatment

Precision medicine applies advanced medical techniques and proper biomarkers aiming at precise location of disease foci and optimal selection of treatment targets to tailor therapy regimen as per deep understanding of disease-specific manifestations and variations as well as individuals’ special conditions with the ultimate objective for the best efficacy. Different from personalized medicine, precision medicine features even more comprehensive considerations covering disease, medication and patient, rooting in more accurate analyses of disease characteristics and manifestation at a deeper level and in emphasizing even better application of medical technology during diagnosis and treatment, as well as in the best appropriateness of drug use. It is a consensus among the medical community that “precision cancer medicine” represents the trend (Mendelsohn, 2013). Predictive biomarkers help clinicians identify the patients with potentials to benefit from certain treatments and enable them make corresponding decision. In oncology, when a biomarker is proved to show statistical difference in therapeutic effect between the two patient populations with the biomarker positive versus negative, it is considered being predictive. For example, human epidermal growth factor 2 (HER2) can serve as a molecular marker, since its expression level in breast cancer, gastric cancer and other tumors predicts the efficacy of HER2 targeted drugs (Swain et al., 2023); among non-small cell lung cancers (NSCLCs), epidermal growth factor receptor (EGFR) deletion of exon 19 and its mutations in exon 21 are predictive for the use of EGFR tyrosine kinase inhibitors, such as osimertinib and erlotinib (Chan and Hughes, 2015). AI also presents some advantages for predictive or prognostic imaging biomarkers, since AI-aided analyses of imaging biomarkers are non-invasive, non-destructive, rapid, easily serializable and cheap, and because they only require conventional radiological scans (Verma et al., 2017), moreover they are fully compatible with existing clinical workflows, just like those of AI-aided pathological biomarkers (Bera et al., 2019). Trebeschi et al. (2019) established and validated a machine learning biomarker capable of predicting immunotherapy responders and non-responders, using AI-supported characterization of the primary and metastatic lesions of advanced malignant melanoma and NSCLC post-immunotherapy; in addition, the biomarker could characterize the tumors on the whole 3D volume, avoiding possible such sampling errors in heterogeneous biopsy samples as those reported in the literature (Cyll et al., 2017), and it was able to detect the changes of tumor microenvironment.

As another contributive approach in precision therapy and a new genetic testing method emergent in recent years, gene sequencing can be used to predict individual health by analyzing the whole sequences of genetic molecules with samples of personal blood or saliva, demonstrating positive effect in human health management. The value of gene sequencing lies in identifying one’s susceptible gene to guide cancer prevention and treatment (Chen et al., 2022). ML-assisted methylation sequencing of circulating tumor DNA provided advantages in cancer screening and treatment efficacy assessment (Fiala and Diamandis, 2019). For the first time, a research team applied DL to detect characteristics of multi-omics relevant to survival of patients with hepatocellular carcinoma (HCC); with their DL-aided model built on RNA sequencing, miRNA sequencing and TCGA methylation data together with clinical data from 360 HCC patients, they achieved prediction performance comparable to that derived from the combination of genomics and clinical data (Chaudhary et al., 2018). Cristiano et al. (2019) established an ML model incorporating genome-wide fragment characteristics to evaluate fragmentation pattern of cell-free DNA in the whole genomic length, realizing sensitivities ranging from 57 to 99% + with the specificity at 98% and an overall area under ROC curve (AUC) of 0.94 among seven types of cancer, including bile duct, breast, colorectal, gastric, lung, ovarian, and pancreatic cancers; in 75% of the cases, fragment characteristics could be used to determine tissues of cancer origin from limited number of foci (Cristiano et al., 2019).

Apart from gene sequencing, immunotherapy constitutes another important part of precision medicine. As to oncology, the main immunotherapy strategies include adoptive cellular immunotherapy, immune checkpoint monoclonal antibody therapy, vaccine therapy, non-specific immune stimulation (cytokine therapy). Despite the impressive progress in immunotherapy, obstacles and challenges are still in the way, including limitation in response rate and inability to predict efficacy and side effects (such as autoimmune response or cytokine release syndrome), thus hindering further application of immunotherapy in clinical practice (Zhang and Zhang, 2020). AI partly overcomes these problems and provides precise treatment by means of, for instance, evaluating efficacy of immunotherapy (Ao et al., 2022), predicting patients’ prognosis and treatment response (Xu et al., 2021; Wang et al., 2022), and distinguishing tumor subtypes to guide treatment (Zhu et al., 2023). Here are some particular examples: plasma cell subtyping with the help of an artificial neural network based on ML algorithm could provide an effective prognostic indicator for prostate cancer and be an option for potential responders to immunotherapy (Xie et al., 2022). With an ML algorithm, a classifier based on five hub gene was constructed to predict the stemness subtypes of patients with hepatocellular carcinoma in a more convenient and applicable way; it could not only act as a guide for further study of the mechanism between tumor stem cells and tumor microenvironment, but also as a potential method for selecting patients more responsive to immunotherapy (Chen et al., 2022). Zhu et al. (2023) used ML methods to identify colon cancer subtypes to provide guidance for subtype-specific medication. Another study integrated ML and single-cell trajectory analysis of T cell depletion to establish a T-cell exhaustion score (TES) model for predicting disease prognosis and immunotherapy responsiveness in patients with colon cancer (Shen et al., 2023). DNA methylation profile has been identified as an indicator of tumor immune microenvironment; because of its stability and convenient measurement via fluid biopsy, it becomes a potential hotspot for predicting therapeutic response to immune checkpoint inhibitors (ICIs). A research team used the methylation level of selected sites to construct an ML model for responsiveness prediction, and this model performed well at both pan-cancer and tumor-specific levels (Xu et al., 2021). A team from Pohang University of Science and Technology proposed a ML framework that uses web-based analysis to identify biomarkers of ICI therapy, and therewith they accurately predicted the ICI response to melanoma, gastric and bladder cancers (Kong et al., 2022). For those patients being sensitive to immunotherapy drugs, ELISE (Ensemble Learning for Immunotherapeutic Response Evaluation) was proposed to generate a highly accurate method for predicting individual responses; the trial results showed that ELISE offered higher accuracy in predicting the response of esophageal adenocarcinoma patients to Atezolizumab; in addition, ELISE could be scaled to accurately evaluate the responsiveness of various immunotherapeutic drugs among other cancers, like programmed death-1/programmed death-ligand 1 (PD1/PD-L1) inhibitors against metastatic urothelial cancer, and melanoma antigen family A3 (MAGE-A3) immunotherapy for metastatic melanoma (Jin et al., 2022).

#### Drug research and development

The history of new drug research and development is also a history of technological evolution thereof, from random drug screening during 1930s to 1960s, across high-throughput selection within 1970s to the early years in 21st century, and to current virtual exploration. Bioanalysis enhanced by ML provides inspires in mining characterization and association in biological networks, supplement to effective deal with high-throughput data of heterogeneous and complex molecules (You et al., 2022). Rapidly developing multi-omic technology in the oncological area has become one of the critical factors for AI-aided biological analysis in discovering new anticancer targets (do Valle et al., 2018). These techniques primarily manifest in the analysis of epigenetics, genomics, proteomics, and metabonomics as well as multi-omic integration. For example, do Valle et al. (2021) have developed a web-based biological analysis framework to calculate the proximity between polyphenol targets and disease proteins; their study showing the network relationship between disease proteins and polyphenol targets provides a calculation method for revealing the effects of polyphenols on disease and offers a basis for finding new anticancer targets (do Valle et al., 2021). A supercomputer provided by International Business Machines Corporation (IBM) enables Atomwise, a US drug research and development company, to screen molecules with potential for drug development from a large-scale data storage system; relying on the AI platform “Interrogative Biology” built by itself, this research company makes an in-depth analysis of the defense system of human cells via the *in vivo* mechanism, and it explores the physiological law of human body with the help of big data and AI technology to find human molecules with therapeutic potential; while controlling costs and saving workload, this mode contributes to cancer therapy as well (Barden and Omuro, 2023). AlphaFold was awarded the 2024 Nobel Prize in Chemistry. AlphaFold AI plays a significant role in drug discovery, particularly in protein structure prediction, target identification, virtual screening, drug design, and understanding mechanisms of drug resistance. First, AlphaFold can efficiently and accurately predict three-dimensional protein structures, which is critical for understanding the functions and interactions of drug targets (Jumper et al., 2021). In addition, by leveraging its structural prediction capabilities, potential drug targets can be identified, especially in research focused on tumors and other complex diseases. The protein structure information provided by AlphaFold can also be applied to virtual screening, aiding in the rapid assessment of compound-target binding affinities. During drug design, its structural data can be utilized for molecular docking and drug optimization, enhancing the efficiency of new drug design (Baek et al., 2021). Finally, by analyzing how mutations affect protein structure, AlphaFold assists in studying mechanisms of drug resistance, offering new insights into managing drug resistance (Zhao et al., 2024). AlphaFold continues to evolve, with AlphaFold 3 (AF3) extending its applications beyond protein structure prediction to encompass high-precision predictions for 59 types of complexes, including the structures and interactions of diverse biomolecules like nucleic acids and ligands. However, AlphaFold 3 still faces limitations in stereochemistry, model hallucination, dynamics, and target-specific accuracy (Abramson et al., 2024).

### Surgical treatment

The application of AI in surgery is represented mainly in surgical decision-making, postoperative prediction and robotic surgery. As to the first aspect, here are some examples: The trial with AI plus multicenter registration data to predict disease-free survival rate among participants with pancreatic cancer demonstrated that AI could provide a valuable decision support system for these patients going to receive surgery (Lee et al., 2021). Neoadjuvant systemic therapy (NAST), though, achieved complete pathological remission in 40–70% of breast cancer patients, the uncertainty of the residual disease still makes it hard to identify patients who are eligible to omit surgery (Heil et al., 2021). This difficulty contributed to the birth of a multimodal ML algorithm (Intelligent Vacuum Assisted Biopsy (VAB)) that integrates four variables: patient, tumor, imaging and biopsy, and this algorithm proved its performance by reliable elimination of cancer if any left from NAST (Pfob and Heil, 2023; Pfob et al., 2022). For early gastric cancer, the biggest challenge in the selection between endoscopic resection and surgical excision is the differentiation of invasion depth as intramucosal or submucosal, but no existing accurate method is available (Sano et al., 1990). To address this problem, an AI classifier was designed and validated, accordingly a diagnostic approach was fabricated involving both AI and endoscopists, and it truly improved the diagnostic efficiency (Goto et al., 2023). In terms of robotic surgery, it brings many advantages for surgeons, like accurate movement, instability resistance, large spatial freedom and potent three-dimensional imaging (Park and Hyung, 2020). For instance, Da Vinci, a robotic surgical system developed by Intuitive Surgical, timely provides doctors with objective and accurate surgical information (Nota et al., 2019). At present, robot-assisted surgery has been used for endometrial cancer (Zanagnolo et al., 2017), thymoma (Shen et al., 2022), gastric cancer (Park and Hyung, 2020), colon cancer (Cuk et al., 2022), prostate cancer, bladder cancer (Falagario et al., 2020) and so on, and it exhibits multiple advantages in both intraoperative and postoperative stages, i.e., less blood loss (Park and Hyung, 2020) and drainage during operation, and less days of pleural drainage, shorter hospital stay and less complications after operation (Shen et al., 2022). Finally, AI is promising in prediction of postoperative recurrence and survival rate. Chung Heewon’s team developed an AI model that used a large training cohort and a number of variables (including preoperative and postoperative nutrition and fat/muscle index) to predict 5-year survival probability of stomach cancer patients at 1 year post-gastrectomy, and they achieved generally accurate prediction (Chung et al., 2023); Lai et al. (2023) collected data from 2,936 patients to build a training set, developed an AI model TRAIN-AI to predict the recurrence of hepatocellular carcinoma after transplantation, and built a network calculator to improve the availability of the model; they planned further update of this AI model by increasing the number of patients (Lai et al., 2023). Provided proper application after strict assessment and external verification, AI may change surgical care by supporting surgical decision-making, identifying and mitigating changeable risk factors, identifying and managing complications, and sharing resource-use decisions (Loftus et al., 2020). However, surgical robots may prolong surgery duration and increase medical costs (Olavarria et al., 2020), requiring attention in further development and improvement.

### Intelligent omics radiotherapy

Before making a radiotherapy plan, doctors may averagely take more than 200 CT images for each patient and spend 3–5 h to identify the target area. AI may lower the workload herein. Specifically speaking, an AI system first defines an imaging scheme proper for a particular confirmed diagnosis, then delineates the target tumor areas on CTs through automatic image recognition, and provides a corresponding radiotherapy plan to submit doctor for manual review; Moreover, AI can monitor the implementation of the plan during actual treatment (Liu and Yu, 2022). The application of AI in radiology has greatly improved the accuracy and safety of every step in cancer radiotherapy, including initial decision-making, treatment planning, and in-process dose adjustment, as well as efficacy evaluation and side effect prediction.

#### Radiotherapy decision-making

Developed by IBM with the help of top oncologists at Memorial Sloan Kettering Cancer Center (MSKCC), Watson for Oncology (WFO) is an AI-aided medical application that focuses on providing decision support in cancer treatment. It uses AI and cognitive computing technology in analyzing medical literature, patient records and other clinical information to help doctors make personalized cancer treatment plans. A meta-analysis evaluated the consistency of treatment regimens between WFO and multidisciplinary team (MDT) involving seven types (breast, rectal, colon, stomach, lung, ovary and cervical) of cancer and concluded that they were highly comparable (Jie et al., 2021). WFO features in improved work efficiency, reduced workload (Printz, 2017), lowered artificial calculation errors in both radiotherapy regimen and drug selection (Keiffer, 2015). It allows more patients participate in decision-making of their own treatment plan and know more about possible adverse effects, therefore being beneficial to alleviating physician-patient disputes (Fang et al., 2018). Nevertheless, due to variation in clinical guidelines, drug preference and economic factors among different countries, the consistency between WFO and MDT for cancer patients is limited in some aspect, showing requirements for further improvement of the system.

#### Delineation of organs at risk (OARs) and target areas

As a new type of radiotherapy, intensity-modulated radiotherapy (IMRT) provides accurate conformal radiation with beams at different intensity based on differentiation tumor target areas from OARs. Contouring or delineation is the premise and guarantee for efficacy and safety of radiotherapy. Manual delineation, the traditional way, is usually time-consuming and tedious, still with significant interobserver variation. So oncological researchers in the field of breast cancer (Mikalsen et al., 2023), prostate cancer (Urago et al., 2021), cervical cancer (Yao et al., 2022) and the like have been exploring the feasibility of clinical utilization of AI in contouring tumor target and OARs. Mikalsen et al. (2023) prospectively included 30 patients who received radiotherapy for breast cancer to evaluate the geometric consistency between automatic segmentation and manual contour. Their verification showed that the profile of OARs generated by the DL segmentation model (DLS) could be used in clinical practice, but most of the clinical target volume (CTV) according to the DLS required correction. Urago et al. (2021) compared MIM Maestro (based on atlas) and MIM Contour Protégé AI (based on artificial intelligence) in automatic segmentation of OARs for 21 patients with prostate cancer. Their work showed that the AI-based model rendered higher accuracy in bladder and rectum than three atlas-based ones: Dice similarity coefficient (DSC), Hausdorff distance (HD) and mean distance to agreement (MDA) (Urago et al., 2021). Yao et al. (2022) studied the application of both AccuLearning-based small sample size training and the AccuContour automatic delineation in MRI imaging of patients with cervical cancer; compared to manual procedure averagely lasting 30 min, AI technique spent only 1 min, and the latter displayed good performance for multiple structures; the result suggested that the smart approach is clinically feasible to improve the quality and efficiency in segmentation of OARs related to radiotherapy against cervical cancer (Yao et al., 2022). Another team tried AccuContour for automatic delineation of OARs in nasopharyngeal carcinoma subject to radiotherapy, and they found that this AI-aided software basically worked well, and that the results of small volume OARs, although with an accuracy slightly poor than that of larger volume ones, could be used in clinical work after minor rectification by doctors (Wu et al., 2021). All these above verified that AI-assisted radiotherapy greatly improves the work efficiency of doctors and enjoys fairly good accuracy. However, some limitations in AI application are also indicated by these studies as follows: (1) small sample size for training; (2) dataset annotation and delineation criteria with variation dependent on local preferences and institutional standards; (3) challenged interpretability and data dependence of the models because of inherent complexity of basic algorithms; and (4) risks of weakening clinicians’ role and losing some basic knowledge due to over-dependence on automation (Batumalai et al., 2020).

## Conclusion

Artificial intelligence has remarkably achieved in medical image analysis and pathological diagnosis. Its deep learning technology enables automatic identification and analysis of patterns from tumor images, and it improves early diagnosis of tumors, contributing to reduced burden of medical system and alleviated shortage of medical resources. In addition, AI stands out for its performance in tumor treatment planning and in provision for doctors with more suitable advice on the ground of accurate analysis of clinical data to formulate individual regimens, and in turn it improves therapy success rate, cancels unnecessary treatment to reduce economic burden of patients, and ultimately it realizes precise medical care. In spite of its great progress in tumor diagnosis and treatment, AI still faces some problems and challenges that may affect its application in practice: (1) sample data imbalance: relatively poor availability of data for some types of tumor resulting in the imbalance of samples used for training AI; (2) data privacy and security issues; (3) poor interpretation: being regarded as “black box” models, complex AI techniques such as deep learning are usually difficult to be explained for their rationale in decision-making. In the field of medicine especially in the diagnosis and treatment of tumors, healthcare professionals and patients prefer to understand the mechanism leading to certain specific diagnosis or treatment recommendations. Therefore, lack of exact interpretation of the models may limit their practical application; (4) challenges in clinical verification; (5) regulatory and ethical issues. To sum up, the application of AI in tumor diagnosis and treatment has brought revolutionary changes in the field of medicine, but still companied by some problems requiring settlement to ensure its effectiveness and reliability in real-world clinical practice.
